# Young-Onset Dementia and Neurodegenerative Disorders of the Young With an Emphasis on Clinical Manifestations

**DOI:** 10.7759/cureus.30025

**Published:** 2022-10-07

**Authors:** Kaynaat Fatima, Ashok M Mehendale, Himabindu Reddy

**Affiliations:** 1 Department of Community Medicine, Jawaharlal Nehru Medical College, Datta Meghe Institute of Medical Sciences, Wardha, IND

**Keywords:** alzheimer's dementia, huntington's disease, parkinson's disease, young-onset dementia, depression

## Abstract

Young-onset dementia (YOD) refers to a neurological ailment primarily affecting people below 65 years of age in roughly about 8% of cases found through various researches. The high rate of prevalence of secondary dementias among older patients proves that younger people show a better prognosis of the conditions causing dementia than older people. However, effective interventions have to be usually provided early in the course of cognitive decline to help facilitate cognitive improvement. The risk of development of prodromal dementia is high if there is a development of psychoses in middle-aged or older people. When there is a development of psychoses in middle to late life, the likelihood of this indicates prodromal dementia is high. The clinical presentation is quite variable and often subtle in frontotemporal dementia (FTD) but may be dominated by personality change, behavioral disturbances, motivation, or the loss of empathy. There is great heterogeneity in the probable causes of dementia in young age as compared to dementia in old age, and some observed differences also exist in the course and characteristics of the disease. These causes may range from the most probable cause such as Alzheimer's disease (AD) to causes with low probability, such as metabolic disorders and prion diseases.

The symptoms of young-onset dementia include a gradual development of personality and behavioral changes over a period of years. However, in the initial stages of young-onset dementia, this change can be attributed to various issues, such as depression, marital problems, and menopause. Other neurodegenerative diseases such as Huntington's disease show presentations such as changes in personality, chorea, and depression that can be observed in patients in their early adulthood. A few other neurodegenerative disorders are myoclonic epilepsy with ragged red fibers (MERRF) and mitochondrial encephalopathy, lactic acidosis, and stroke-like episodes (MELAS) with presentations such as characterized muscle weakness, poor growth, problems with vision and hearing, and the involvement of the multi-organ system, including the central nervous system to name a few. There is also the prevalence of juvenile parkinsonism in the community, which represents a group of clinicopathological entities present before the age of 21. Young-onset Parkinson's disease (PD) (YOPD) appears to have the same pathological presentation as late-onset Parkinson's disease (LOPD). Recent researches have proved that "gene therapy" can be useful in the treatment and in preventing the progression of symptoms in cases of neurodegenerative diseases.

## Introduction and background

The World Health Organization reported that 10 neurological illnesses have been found and are stated to "represent a large component of the global burden of the neurological disorders, namely, epilepsy, headache disorders, dementia, neuroinfection, Parkinson's disease, traumatic brain injury, multiple sclerosis, stroke, pain associated with neurological disorders, and neurological disorders linked with malnutrition" [1].

The term "young-onset dementia" (YOD) refers to a neurological illness that primarily affects people under 65 years of age [2]. Young-onset dementia is acknowledged as a significant social and clinical issue that has devastating effects on both the patient and the caregiver [3]. A wide range of mental, behavioral, cognitive, or neurological symptoms may appear in young-onset dementia [3]. The economic and psychosocial consequences are often quite severe, particularly for patients belonging to the younger age-group [4]. Clinicians face major diagnostic challenges; therefore, a rapid diagnosis is crucial for patient management and counseling [4]. Young-onset Parkinson's disease (YOPD) patients first experience symptoms between the age of 21 and 40 [5]. Juvenile parkinsonism develops before the age of 21. In contrast, young-onset Parkinson's disease (YOPD) develops between the ages of 21 and 40, as previously indicated, though other researchers have also suggested that the age of 50 is the cutoff [5]. As it affects patients in the prime years of their life, it often has an extraordinary impact on their profession, family, and social life [6].

Phenotypical and genotypical differences have been identified between young-onset Parkinson's disease (PD) and late-onset Parkinson's disease (LOPD) [6]. Alzheimer's disease (AD) was initially described as a neurodegenerative ailment that manifested in early or middle age; if it starts before the age of 65, it is referred to as early-onset AD [7]. Originally, Alzheimer's disease (AD) meant a disorder having an early onset (early-onset Alzheimer's disease: <65 years of age) and, therefore, did not include any older patients with "senile dementia." The first patient, Auguste Deter (1850-1906), reported this neuropathology that showed the onset of symptoms in her late 40s before she was diagnosed with dementia at the age of 51 [8]. The symptoms portrayed by her included confusion, memory loss, language impairment, and aggressive, agitated and paranoid, and unpredictable behavior, and on autopsy, the most important finding was that she had the characteristic neuropathological marker of Alzheimer's disease, now recognized as the extracellular amyloid-positive neuritic plaques along with intracellular tau-positive neurofibrillary tangles (NFT) [8]. An advanced method for treating the various neurodegenerative diseases is "gene therapy," as it has the potential to provide therapeutic benefit to the millions of people suffering from neurodegenerative diseases through numerous means, including symptom control, neuroprotection, the direct correction of pathogenic mechanisms, and neurorestoration [9]. Therefore, the therapeutic efficacy depends on knowledge pathogenesis of the disease and the required spatial and temporal specificity of gene expression [9].

## Review

Dementia

Causes

The most significant group of this condition's causes is likely its reversible ones, which include viral, inflammatory, metabolic, and toxic etiologies. Blood test results, additional symptoms, cerebrospinal fluid (CSF) analyses, characteristic findings on neuroimaging, tissue biopsies, and neurophysiology tests are differentiating factors among the neuropathologies [2]. Keeping the first perspective under consideration, there is great heterogeneity in the probable causes of dementia in young as compared to dementia in old individuals, and there are observed differences in the characteristics and course of the disease. These causes may range from the most likely cause such as Alzheimer's disease to less likely causes, such as prion diseases and metabolic disorders [3]. A recognizable emotional trigger, a lack of advancement over time, and an abrupt onset are among the presenting signs and symptoms that frequently point toward a psychiatric condition rather than a neurodegenerative diagnosis. Periodic clinical reviews also aid in defining this difference. The prognosis and treatment of early-onset dementia may be impacted by an accurate and possibly early diagnosis [3]. Several YODs have hereditary manifestations, and genetic testing, which is becoming increasingly popular as a technique of diagnosis confirmation and aids in assessing the risk in the family, is one way to determine the condition [10].

Types

Compared to late-onset dementia (LOD), a smaller percentage of cases of young-onset dementia shows the etiology to be a neurodegenerative illness. Even though dementia of Alzheimer's disease (AD) is the most prevalent kind of dementia among young-onset dementia (YOD), the percentage of cases attributable to AD in YOD is much lower (15%-40%) than it is in late-onset dementia (LOD) (50%-70%) [11]. However, there are not many AD cases in people under the age of 45, and most of these cases are unusual because they are caused by autosomal dominant familial Alzheimer's disease [12]. Among the other degenerative dementias, Huntington's disease and frontotemporal dementia (FTD) are more prevalent in the younger population, while Parkinson's dementia and dementia with characteristic Lewy bodies are perhaps less commonly seen. Although the reported proportion of patients with vascular dementia as the etiology varies widely, it is more likely to exhibit similarities to LOD [11,13,14].

Young-onset dementia is more frequently accompanied by secondary dementias (approximately 20% of cases), which can include multiple sclerosis dementia, alcohol-related dementia (ARD) that accounts for 5%-10% of cases, dementia brought on by the human immunodeficiency virus (HIV), traumatic brain injury, and a variety of metabolic, neoplastic, viral, and immunological illnesses, many of which are exceedingly rare and may occasionally be accompanied by genetic alterations [13]. The high proportion of patients with secondary dementia proves that younger people show a better prognosis of the conditions causing dementia than older people. However, effective interventions have to be usually provided early in the course of cognitive decline to help facilitate cognitive improvement. Examples include treating cerebral vasculitis using steroid therapy and decreasing alcohol consumption in addition to thiamine replacement in ARD [13]. There are several "dementia plus" syndromes, most of which are classified as secondary dementias and frequently manifest with various physical problems [14]. The following table represents some common "dementia plus" syndromes [14].

**Table 1:** List of common "dementia plus" syndromes CNS: central nervous system [14]

Types of dementia	Physical syndromes
Parkinson's dementia	Parkinsonian syndromes
Dementia with Lewy bodies	Seizures, parkinsonian syndromes, and dysautonomia
Vascular dementia	Parkinsonian syndromes, seizures, and pyramidal syndromes
Progressive supranuclear palsy	Gaze palsy and parkinsonian syndromes
Frontotemporal dementia	Parkinsonian syndromes, respiratory failure, and pyramidal syndromes
Huntington's disease	Chorea, parkinsonian syndromes, and dystonia
Corticobasal degeneration	Chorea, dystonia, and parkinsonian syndromes
Alcohol-related dementia	Peripheral neuropathy, hepatic disease, seizures, and ataxia
HIV-related dementia	Opportunistic infections, skin lesions, and peripheral neuropathy
Multiple sclerosis	Pyramidal syndromes and ataxia
Traumatic dementia	Parkinsonian syndromes
Cerebral vasculitides (including CNS lupus)	Impairment of vision, headache, focal neurological signs, and skin lesions
Normal pressure hydrocephalus	Ataxia and urinary symptoms

Clinical Presentations

Clinical manifestations in young-onset dementia (YOD) vary more than in late-onset dementia (LOD), partly because of the distinction in the profile of dementia types. While objective and subjective memory complaints are significant characteristics, additional frequent presentations in "dementia plus" disorders include behavior change, depression, and physical symptoms such as gait difficulty, seizures, vision impairment, and peripheral neuropathy [11]. As Alzheimer's disease is the most common type of young-onset dementia, particularly after crossing the fifth decade of life, the typical presentation of late-onset Alzheimer's disease includes the representation of gradually progressive impairment of short-term memory and word-finding difficulties, and disorientation also occurs in young-onset dementia. Due to insufficient clinical assessment, people may first be reassured that nothing is wrong despite having subjective cognitive symptoms [14]. The presence of persistent depression in numerous types of young-onset dementia is a typical pre-dementia diagnosis made by many specialists, and this finding is not surprising given the fact that there is a presence of major depressive symptoms in a large proportion of patients with young-onset dementia [15].

In some instances, what is initially diagnosed as depression turns out to be social withdrawal and apathy due to the impairment of the executive function of the frontal lobe [16]. Personality and behavioral abnormalities are typical early symptoms of several kinds of young-onset dementia, including alcohol-related dementia (ARD) and frontotemporal dementia (FTD), where early frontal lobe impairment is frequently observed. Social withdrawal, apathy, obsessive conduct, sexually inappropriate behavior, and changing eating patterns are a few behavioral changes [17]. There is a gradual development of the personality and behavioral changes over a few years, and during the early stages of YOD, a range of conditions, including depression, marital conflicts, menopause, alcohol abuse, or job stress, can be blamed for this change. The cognitive deterioration, which may be reversible in certain cases, may go unnoticed in those with a long history of alcohol addiction. Their functional impairment may be mistakenly attributed to self-neglect, sadness, or chronic intoxication [17].

When there is a development of psychoses in middle to late life, the likelihood of this indicates prodromal dementia is high [18]. The clinical presentation is quite variable and often subtle in frontotemporal dementia but may be dominated by personality change, behavioral disturbances, motivation, or the loss of empathy [19]. Hyperorality and the development of a sweet tooth are identifying traits, while magnetic resonance imaging (MRI) frequently reveals excessive frontal lobe atrophy, which is often mild [19]. Due to the loss of semantic knowledge about the meanings of words and objects, semantic dementia mimics progressive fluent aphasia, resulting in grammatically accurate speech. Still, it is progressively circumlocutory and vacuous [19].

Diagnosis

The extensive presentations and a low expectation that the underlying cause at an early age might be dementia result in a long time for diagnosis compared to that in late-onset dementia. Thus, there are frequent complaints about the diagnostic delay from the caretakers [20]. The extent to which the duration of diagnosis is extended is due to delays caused by misdiagnosis [20]. There are certain clinical scenarios in patients of middle age that should be alarming for the clinician and indicate performing a complete formal assessment of cognitive functions with the aid of an instrument used for standardized cognitive screening. The following should be obtained as auxiliary history for reassurance from the patient's friends or family: close family members who express concern about the patient's cognitive function; heavy substance abuse or alcohol abuse of a duration of five years or more; treatment-resistant anxiety or depression, particularly if there are complaints of subjective cognitive impairment; HIV-positive status of the patient; family history of YOD; behavior change inconsistent with the patient's premorbid personality; a progressive neurological disease; chronic systemic diseases, which are known causes of dementia; and patients presenting with thinking concerns or memory loss, particularly if there is a family history of young-onset dementia; a complete and careful neurological examination should be performed by the clinician with special attention to the cerebellar, pyramidal, and extrapyramidal signs [21].

Clarifying the impaired cognitive domain of cognition requires formal neuropsychological testing and bedside assessment of cognitive processes using screening tools such as the Montreal Cognitive Assessment or Mini-Mental State Examination. The development of diagnostic hypotheses connected to the assumed fundamental neuroanatomy is aided by the combination of historical information, behavioral and cognitive characteristics, neurological examination findings, and historical studies [22]. Additionally, this "dementia plus" algorithm offers a helpful framework for clinically evaluating a case of young-onset dementia and has been supported by other experts in the field [23].

Laboratory Investigations

Blood tests may diagnose autoimmune diseases, toxic or metabolic encephalopathies, and infectious etiologies such as syphilis or human immunodeficiency virus (HIV). According to the recommendations of a professional organization, all patients with YOD should be required to undergo neuroimaging (most preferably magnetic resonance imaging {MRI}) and, if practical, cerebrospinal fluid (CSF) analysis [24]. Brain atrophy patterns and MRI sequences showing signal changes are quite helpful in reducing the differential diagnosis. The addition of MRI may aid in detecting areas of hypometabolism in patients with little alterations on fluorodeoxyglucose-positron emission tomography (FDG-PET) imaging. CSF analysis may make it easier to pinpoint inflammatory or viral causes of young-onset dementia. Electromyography (EMG), nerve conduction studies (NCS), and electroencephalography (EEG), among other neurophysiology tests, can help show related myopathy, neuropathy, and seizure activity, respectively. Additionally, a tissue biopsy may be helpful in the identification of lysosomal storage disorders or leukodystrophies using an enzyme test of leukocytes or skin fibroblasts, as well as mitochondrial disorders via muscle biopsy. Although rarely used, cerebral biopsy has shown to be a generally safe and effective way to diagnose dementia. It should be considered if there is even a remote possibility that the condition may be curable [24].

Other neurodegenerative diseases

A mutation of autosomal dominant nature on chromosome 4 with cytosine, adenine, and guanine (CAG) trinucleotide chain repeats in the *Huntingtin* (*HTT*) gene, resulting in Huntington's disease [25]. The presentations such as changes in personality, chorea, and depression can be seen in patients in their early adulthood [26]. Myoclonic epilepsy with ragged red fibers (MERRF), Kearns-Sayre syndrome, and mitochondrial disorders, including mitochondrial encephalopathy, lactic acidosis, and stroke-like episodes (MELAS), are rare disorders characterized by muscle weakness, poor growth, problems with vision and hearing, and the involvement of the multi-organ system, including the central nervous system [27,28]. Juvenile parkinsonism represents a group of clinicopathological entities present before the age of 21; young-onset Parkinson's disease (YOPD) appears to be the same pathological presentation as late-onset PD [29]. The classical clinical features of Parkinson's disease consist of cogwheel rigidity, resting tremor (usually present as unilateral), bradykinesia, and sometimes gait instability and postural reflexes, which are compromised [30]. A positive response to levodopa shows a sign that it's 100% sensitive but not completely specific [30]. The presenting symptoms of young-onset Parkinson's disease show similarity with the classic symptoms of late-onset Parkinson's disease (LOPD). Still, some clinical features appear more eminent in young-onset Parkinson's disease [30]. Paresthesias were present in about 20.5% of patients with young-onset Parkinson's disease compared to 2% of the patients with late-onset Parkinson's disease [31]. It was found that the association of mortality rate with young-onset PD is as compared with the normal population. Still, there is no statistical variation when the comparison is made with other patients with PD [32].

Patients with an autosomal dominant familial pattern of Alzheimer's disease (AD) have a higher risk of passing the disease down to their relatives. They may exhibit atypical clinical symptoms, such as myoclonus, headaches, seizures, pseudobulbar palsy, hyperreflexia, or abnormal gait [33]. Patients with early-onset Alzheimer's disease are more likely to have a history of traumatic brain injury, which has been linked to dementia risk [34]. Researchers have discovered that people with early-onset AD have fewer circulatory disorders, cerebrovascular risk factors, obesity, and diabetes mellitus than patients with late-onset AD [35,36].

Neurodegenerative changes related to altered sleep patterns and COVID-19

The neurodegenerative changes linked to insufficient sleep may be caused by the accumulation of debris in the brain and changes in brain microstructure [37]. To completely comprehend the connection between sleep duration and cognitive decline, as well as the biochemical changes that underlie the association, more data is required. A potentially modifiable risk factor for the prevention of cognitive decline and neurocognitive diseases is sleep quality measures such as sleep length [37]. The etiology of coronavirus disease 2019 (COVID-19) neurological impairment is currently being researched, and there are many different potential explanations. Based on information from other coronaviruses, clinical observations, animal research, and radiographic imaging, the postulated etiologies are supported by this data. For instance, neurological symptoms have been linked to Middle East respiratory syndrome (MERS) and severe acute respiratory syndrome coronavirus (SARS-CoV), which have also been found in the brain and cerebrospinal fluid (CSF) tissue [38]. At least one neurological symptom has been recorded in up to 90% of COVID-19 patients [39]. At a three-month follow-up, even individuals who had no neurological symptoms were showing alterations in their neurological microstructure, indicating that the impacts of neurological conditions may be considerably broader than originally believed [40].

## Conclusions

This article focuses on patients with young-onset dementia, as well as those who care for them who have different needs than older patients. Following diagnosis, the young-onset dementia key worker should be referred to and should take swift action to be taken to establish effective community services and psychological treatment strategies. Acetylcholinesterase inhibitors such as galantamine, donepezil, and rivastigmine may relieve Alzheimer's disease symptoms but may slow the disease's progression. Because YOD patients frequently lack insight and exhibit impairment of judgment, it is crucial to evaluate their level of safety. Environmental alteration, such as exercise or other activities that divert attention, limiting access to food, and using dangerous equipment, frequently plays a significant impact. Occupational and speech therapists can help with other daily tasks and alternate forms of communication. Acknowledging the caregiver burden is crucial when delivering non-pharmacological treatment for these diseases. As new insights into essential aspects of the underlying molecular pathophysiology are gained, therapeutic approaches for gene repair or addition in neurodegenerative brain pathologies continue to change. Amantadine addition can be used to treat levodopa-induced dyskinesias. When patients require extra symptomatic control or are dealing with severe motor problems while receiving pharmaceutically optimized therapy, surgery may be an option. YOPD and late-onset Parkinson's disease are quite similar in many aspects, but YOPD needs special consideration since it differs from late-onset Parkinson's disease sufficiently to need a different strategy for therapy. According to the available data, Parkinson's disease patients who are younger at the time of onset have slower disease progression, more dystonia at the time of onset, less dementia, and more dyskinesias in response to levodopa treatment. It is made easier to comprehend the neurobiology of early-onset AD, as well as the specific differences and challenges associated with managing these patients and assisting their caregivers and family when early-onset AD and its variants are recognized as a particular group of dementias.
